# Outcomes of a Modified Portal Venovenous Bypass Technique During Liver Transplantation in High‐MELD Recipients

**DOI:** 10.1111/ctr.70703

**Published:** 2026-09-29

**Authors:** Michael G. Megaly, Daniel C. Waller, David Matthews, Andrew Adams, Timothy L. Pruett, Karthik Ramanathan, Raja Kandaswamy, Erik B. Finger, Hannah Sutherland, Vanessa Humphreville, Srinath Chinnakotla

**Affiliations:** ^1^ Division of Transplantation, Department of Surgery University of Minnesota Minneapolis Minnesota USA; ^2^ Division of Biostatistics and Health Data Science University of Minnesota School of Public Health Minneapolis Minnesota USA; ^3^ University of Minnesota Physicians Minneapolis Minnesota USA

**Keywords:** liver transplant, model for end‐stage liver disease, portal venovenous bypass

## Abstract

**Background:**

Although portal (venovenous) bypass (pVVB) is uncommon in liver transplantation (LT), it may provide some advantages, particularly for high‐acuity recipients.

**Methods:**

To examine its use in high‐risk patients, we retrospectively evaluated outcomes of a modified pVVB technique among adult LT recipients at a single center, stratified by MELD score (≥35 vs. <35). Primary outcomes were patient and graft survival evaluated withKaplan–Meier estimates and multivariable Cox regression; secondary outcomes included biliary complications, transfusion requirements, viscoelastic parameters, laboratory trends, length of stay, readmission, and need for dialysis.

**Results:**

Between January 2016 and July 2025, 548 patients underwent LT with pVVB; 111 (20.3%) had MELD ≥35. Despite greater baseline severity, higher transfusion requirements, and longer postoperative ICU stay, MELD ≥35 was not associated with increased risk of graft failure (HR 1.19; 95% CI 0.72–1.96), mortality (HR 0.86; 95% CI 0.46–1.61), or biliary complications (HR 1.02; 95% CI 0.65–1.60). Post‐reperfusion viscoelastic measures were similar across groups. Dialysis was more frequent before and after LT in MELD ≥35. No complications or deaths were attributable to pVVB.

**Conclusion:**

Among LT recipients treated with pVVB, MELD ≥35 was not significantly associated with graft failure, mortality, or biliary complications. These findings support the feasibility of pVVB in high‐acuity recipients.

**Abbreviations**
ALT: alanine aminotransferase;
AST: aspartate transaminase
BMI: body mass index
FFP: fresh‐frozen plasma
ICU: intensive care unit
INR: international normalized ratio
LT: liver transplant(ation)
MAC: multi‐access catheter
MELD: model for end‐stage liver disease
PRBC: packed red blood cells
pVVB: portal venovenous bypass

## 1 Introduction

Liver transplantation (LT) remains the cornerstone therapy for patients with end‐stage liver disease [1]. As annual LT volumes continue to rise and donor organs remain scarce, preserving graft function remains essential. Although technically complex, orthotopic LT is generally well tolerated; however, many candidates present with significant portal hypertension and coagulopathy, both of which increase bleeding risk and intraoperative instability.

The severity of liver disease is commonly quantified using the Model for End‐Stage Liver Disease MELD‐Na score. Numerous studies have demonstrated that higher MELD scores are associated with increased perioperative complications in LT [2, 3, 4, 5]. Although the exact threshold defining “high MELD” varies across studies, higher MELD scores have consistently been associated with greater operative complexity, increased intraoperative blood product utilization, prolonged postoperative intensive care unit (ICU) stays, higher mortality, and lower graft survival [2, 3, 4, 5].

During native hepatectomy and graft implantation, clamping of the portal vein and inferior vena cava reduces venous return and causes splanchnic congestion, predisposing to hemodynamic collapse during the anhepatic phase of LT and post vascular anastomosis upon unclamping, as well as bowel edema and bacterial translocation [6]. To mitigate these effects, venovenous bypass (VVB) was introduced to decompress the portal and systemic venous beds while maintaining cardiac preload during the anhepatic phase.

Foundational canine experiments by Moore and Starzl in the 1960s first established this concept [7]. Early clinical adaptations involving systemic heparinization and venoarterial bypass were subsequently abandoned due to poor outcomes [8]. In 1983–1984, several groups implemented a simplified extracorporeal circuit employing heparin‐bonded tubing and low‐shear propulsion to decompress the venous system while minimizing blood trauma [9, 10]. Subsequent clinical series demonstrated improved intraoperative hemodynamic stability, enhanced visceral perfusion (particularly of the kidney and intestine), reduced transfusion requirements, prolonged anhepatic tolerance to facilitate complex hepatectomy or implantation, and improved short‐term survival [9, 11, 12].

Despite these physiologic advantages, routine use of VVB declined with the advent of piggyback techniques and advances in anesthesia, hemodynamic monitoring, and volume management. Additional concerns, including cost, the risk of large‐bore cannulation, circuit‐related complications, hypothermia, and groin and neck wound complications further limited its application [6]. Consequently, many institutions have abandoned its routine use. Evidence remains mixed: a recent Cochrane review found no support for universal application of VVB in all cases [13]. Today, most centers employ VVB selectively for cases involving severe portal hypertension, challenging caval anatomy, or anticipated hemodynamic fragility. For instance, in a 2024 series of 900 LTs, approximately 3% underwent VVB; these cases required greater intraoperative packed red blood cell transfusion but showed no difference in length of stay, acute kidney injury, primary graft dysfunction, or long‐term graft survival, although a higher rate of postoperative non‐anastomotic biliary strictures was observed [14]. Another program with 163 VVB cases advocated selective use to expand acceptance of marginal grafts per national criteria [15].

At our institution, we utilize a modified portal venovenous bypass (pVVB) technique that provides portal decompression without the systemic venous cannulation This technique is simple to use, adds minimal time to the procedure, and averts the complications related to placement of large‐bore cannulas in the neck and wound complications in the groin while effectively decompressing the portal system. In this study, we evaluated the use of pVVB in LT among patients stratified by preoperative risk, defined by a MELD‐Na score ≥35 versus <35. We assessed patient and graft survival, readmission, and biliary complications, alongside intraoperative and postoperative outcomes Through this large single‐center experience, we sought to characterize the use and safety of pVVB across a range of pretransplant disease severity, with particular attention to high‐MELD recipients.

## 2 Methods

We conducted a retrospective cohort study of adult LT recipients who underwent LT with pVVB at the University of Minnesota from January 1, 2016 to July 30, 2025. Patients were stratified by MELD‐Na score ≥35 versus <35, with analyses focused on the association of study outcomes with the recipient's MELD‐Na classification documented on the day of admission for LT. pVVB was routinely employed by five of the six liver transplant surgeons at our institution each of whom used pVVB consistently rather than selectively. use pVVB in all cases.

We selected the MELD threshold of ≥35 based on established national allocation precedent. The Share 35 policy implemented by the United Network for Organ Sharing prioritized candidates with MELD ≥35due to their high acuity, which approached that of status 1A candidates. Additionally, prior studies demonstrate a disproportionate rise in short term mortality beyond MELD 35, supporting its use as a clinically meaningful high‐acuity cutoff [4].

### 2.1 Technique of Portal Venous Bypass

At our institution, pVVB is performed using a closed extracorporeal circuit (Bio‐Medicus, Medtronic, Minneapolis, MN) primed with crystalloid solution and assembled with inflow and outflow cannulas connected to a centrifugal pump. Our LT technique is described in detail elsewhere [16]; briefly, after the abdomen is opened, the falciform ligament, hepatic artery, and bile duct are divided. The portal vein is divided at the hepatic hilum close to the liver using a vascular stapler. Portal inflow to the bypass circuit is established by inserting a 16‐Fr cannula into the portal vein, with return through the right internal jugular vein via a multi‐access catheter (MAC) line placed by anesthesiology (routinely on all LT) (Figure 1). No systemic heparin is given except for patients with known hypercoagulability or normal coagulation, who receive 5000 units IV heparin. The circuit is de‐aired, and flow is gradually increased to achieve 1 L/min, maintaining stable central venous and mean arterial pressures. This technique is generally achieved within a few minutes to connect the MAC line (which is placed routinely by anesthesia in all LT) to the portal cannula placed by the operating surgeon. Upon completion of hepatectomy and caval anastomosis (bicaval or piggyback), bypass flow is stopped, the portal cannula is removed, and portal anastomosis is performed to reperfuse the liver. Following this, the arterial and biliary anastomoses are performed, followed by abdominal closure. There were no contraindications for applying pVVB; it was utilized in all patients in the current study.

**FIGURE 1:**
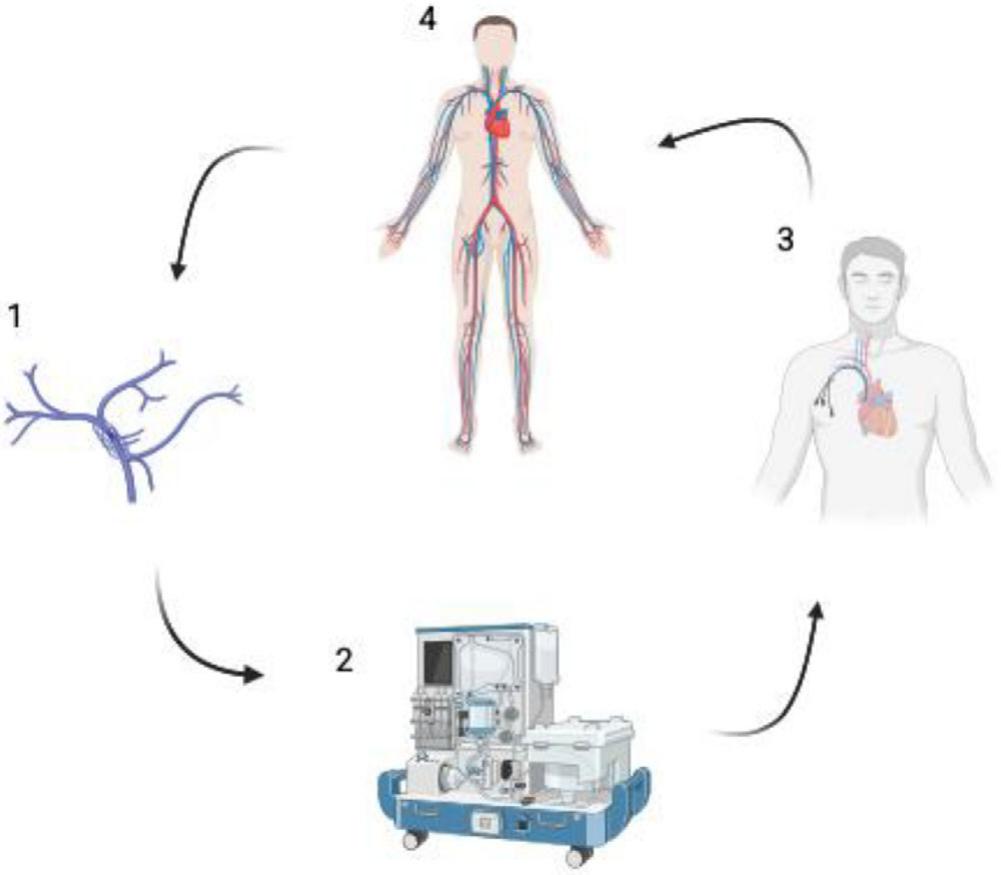
Schematic of pVVB technique. Diagram showing the configuration of portal bypass during orthotopic liver transplantation. The portal vein is accessed in the standard open fashion and cannulated with a 16‐Fr cannula. Portal blood is diverted through an extracorporeal bypass circuit and returned to the systemic circulation via a right internal jugular MAC line. This technique decompresses the splanchnic and systemic venous beds during the anhepatic phase and helps maintain hemodynamic stability.

### 2.2 Outcomes

Primary outcomes analyzed in the study were patient and graft survival. Secondary outcomes included biliary complications (defined as biliary strictures or bile leak); vascular complications; length of stay (LOS); readmission; intraoperative measures including use of blood products (packed red blood cells (PRBCs), fresh frozen plasma (FFP), platelets, cryoprecipitate); and clotting using the Quantra Hemostasis Analyzer (HemoSonics, Charlottesville, VA) (point‐of‐care system that evaluates coagulation by measuring viscoelastic properties), postoperative laboratory trends (hemoglobin, INR, creatinine, bilirubin, AST, and ALT at postoperative days 1,3,7, and 30), and dialysis at postoperative days 7 and 28.

For the primary outcomes of graft survival, biliary complication, and death, survival was estimated with Kaplan‐Meier estimators. Adjusted analyses of associations between primary outcomes and MELD scores ≥35 versus < 35 were conducted with Cox regression models that, for all variables, were adjusted for age, sex, race, body mass index (BMI), diabetes, year of transplant, cold ischemia time, warm ischemia time, donor age, donor status (DCD vs. DBD), dialysis up to 1 week before transplant, and occurrence of acute liver failure as recorded at time of transplant. An exploratory analysis of all other outcome variables was performed, with t‐tests for continuous variables and chi‐squared tests for categorical variables. Adjustments to significance thresholds due to multiple testing were made using the Benjamini‐Hochberg procedure, adjusting for 42 total tests. Significance thresholds were adjusted per the Benjamini‐Hochberg procedure, but only the difference in hemoglobin at 1 day post‐op between study groups would be regarded as non‐significant after these adjustments. All other *p* values below 0.05 are considered significant after this adjustment. Some missingness was present in cold ischemia time, warm ischemia time, and BMI data (5, 6, and 21 missing data points, respectively, out of *N* = 548); this was addressed with median imputation.

Statistical significance for primary outcomes was set at a two‐sided α = 0.05; significance for outcomes in the exploratory analysis was based on Benjamini‐Hochberg adjustments (less than 0.05). Analyses were performed with R, version 4.5.0.

This study was approved by the University of Minnesota IRB (STUDY00012534). Research was conducted in accordance with both the Declarations of Helsinki and Istanbul.

## 3 Results

### 3.1 Patient Characteristics

From January 2016 to July 2025, 796 patients underwent LT, of whom 548 (68.8%) underwent transplantation with pVVB and comprised our study cohort. Of these, 111 (20.3%) had MELD ≥35 and 437 (79.7%) had MELD <35 (Table 1). Mean (SD) MELD for the non‐pVVB was 23.4 (10.9) while the pVVB was 25.2 (10.5). Recipients with MELD ≥35 were younger (50.7 ± 12.4 years vs. 55.0 ± 11.1 years) and had a higher BMI (30.4 ± 6.6 kg/m^2^ vs. 28.7 ± 5.9 kg/m^2^). Sex distribution was similar (female: 43.2% vs. 37.1%). 75.7% of MELD ≥35 were white / non‐hispanic, compared to 82.8% of MELD <35. 26.1% of MELD ≥35 recipients had a diabetes diagnosis prior to transplant, compared to 34.8% of MELD <35 recipients. Acute liver failure was significantly more common among MELD ≥35 patients (18.9% vs. 2.3%).

**TABLE 1:** Baseline characteristics of liver transplant recipients stratified by MELD ≥35 versus MELD <35.

		MELD score		
Variable	Level	35 and over	Less than 35	*p*
Number of Patients		111	437	
Age at Transplant, (mean (SD))		50.7 (12.4)	55.0 (11.1)	<0.001
Sex, *n* (%)	Female	48 (43.2)	162 (37.1)	0.44
	Male	63 (56.8)	274 (62.7)	
	Unknown	0 (0.0)	1 (0.2)	
Race, *n* (%)	Asian	8 (7.2)	30 (6.9)	0.23
	Black	11 (9.9)	20 (4.6)	
	Other	7 (6.3)	20 (4.6)	
	Unknown	1 (0.9)	5 (1.1)	
	White	84 (75.7)	362 (82.8)	
BMI before Transplant, (mean (SD), kg/m^2^)		30.4 (6.6)	28.7 (5.9)	0.008
Type of Transplant, *n* (%)	Li	104 (93.7)	412 (94.3)	0.99
	SLiK	7 (6.3)	25 (5.7)	
Living/deceased donor, *n* (%)	DBD	109 (98.2)	375 (85.8)	0.001
	DCD	2 (1.8)	57 (13.0)	
	Living Donor	0 (0.0)	5 (1.1)	
Diabetes before Transplant, *n* (%)		29 (26.1)	152 (34.8)	0.11
Dialysis, 1 Week pre‐op, *n* (%)		68 (61.3)	61 (14.0)	<0.001
Acute Liver Failure, *n* (%)		21 (18.9)	10 (2.3)	<0.001
Use of OCS, *n* (%)		2 (1.8)	35 (8.0)	0.034
CRRT, *n* (%)		72 (64.9)	97 (22.2)	<0.001
Cold Ischemia Time, median [IQR], min		382.0 [327.5, 450.5]	401.0 [313.0, 504.0]	0.20
Warm Ischemia Time, median [IQR], min		53.0 [41.0, 77.5]	55.0 [42.0, 76.0]	0.87

*Note:* Demographic and preoperative characteristics of 548 liver transplant recipients stratified by MELD score. Continuous variables are expressed as *mean ± SD*, and categorical variables as *n (%)*. Statistical comparisons were performed using Student's *t*‐test or Mann–Whitney *U* test for continuous data and the chi‐square test for categorical variables. Unadjusted *p* values.

Abbreviations: BMI, body mass index; CRRT, continuous renal replacement therapy; DBD, donation after brain death; DCD, donation after circulatory death; OCS, Organ Care System; SD, standard deviation; SLiK, Simultaneous Liver/Kidney.

### 3.2 Surgical Characteristics

Rates of liver‐alone and simultaneous liver–kidney (SLK) transplantation were comparable between MELD strata (93.7% vs. 94.3%). MELD ≥35 recipients were more likely to receive donation‐after‐brain‐death (DBD) grafts (98.2% vs. 85.8%) and less likely to receive donation‐after‐circulatory‐death (DCD) grafts (1.8% vs. 13.0%) or living‐donor grafts (0% vs. 1.1%). Use of the Organ Care System (OCS) was less frequent (1.8% vs. 8.0%). Median [IQR] cold ischemia time for MELD ≥35 was 382 [328, 451] min, compared to 401 [313, 504] min for MELD <35; warm ischemia time for MELD ≥35 was 53 [41, 78] min, compared to 55 [42, 76] min for MELD <35. A summary of differences is found in Table 1.

### 3.3 Donor Characteristics

The average age of donors for MELD ≥35 recipients was 42.2 years, compared to 46.4 years for MELD <35. 60.4% of the donors for MELD ≥35 recipients were male, compared to 62.9% of MELD <35 recipients. Donors had no statistically significant differences between groups across lab values of BMI, sodium, creatinine, AST, ALT, or bilirubin.

### 3.4 Primary Outcomes

Patient and graft survival were similar between the MELD cohorts. MELD ≥35 was not found to be significantly associated with the risk of graft failure (HR: 1.19; CI: 0.72–1.96; *p* = 0.49), nor was mortality (HR: 0.86; CI: 0.46–1.61; *p* = 0.64). However, the above confidence intervals are wide and should therefore be interpreted with caution. Kaplan‐Meier curves of graft failure and patient mortality are shown in Figure 2.

**FIGURE 2:**
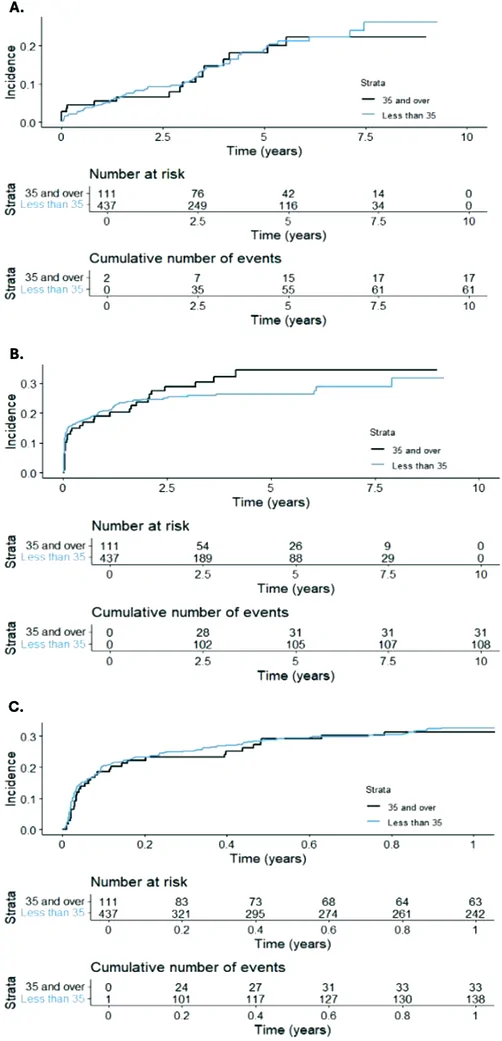
Kaplan–Meier curves for post‐transplant outcomes. Kaplan–Meier curves comparing (A) patient survival, (B) graft survival, and (C) biliary complication‐free survival between the cohorts with MELD ≥35 and MELD <35. Events over 10 years are shown for A and B; for C, all events occurred within 1 year. Survival estimates were compared using the log‐rank test. No statistically significant differences were observed between groups. Abbreviation: MELD, model for end‐stage liver disease.

Causes of Death:

Of the 78 total deaths recorded amongst the transplant recipients, the causes were as follows: malignancy (*n* = 20, 25.6%), infection (*n* = 13, 16.7%), respiratory failure (*n* = 10, 12.8%), cardiovascular cause (*n* = 7, 9.0%), graft failure (*n* = 2, 2.6%), cerebrovascular (*n* = 5, 6.4%), hemorrhage (*n* = 2, 2.6%), suicide (*n* = 1, 1.3%), other (*n* = 16, 20.5%), and unknown (*n* = 2, 2.6%).

Three intraoperative deaths occurred: two among recipients with MELD ≥35 and one among recipients with MELD < 35. One patient with MELD ≥35 was undergoing a third liver transplant requiring extensive adhesiolysis and experienced massive blood loss, progressive coagulopathy and acidosis, and escalating vasopressor requirements despite massive transfusion. Following reperfusion, the patient developed acute right heart diffuse coagulopathy and an atrial thrombus. The second patient with MELD ≥35 was found to have necrotic / hemorrhagic pancreatitis leading to shock. The recipient with MELD <35 developed refractory non‐surgical coagulopathy, followed by hemorrhagic shock and cardiopulmonary collapse. None of these deaths were clearly attributable to pVVB.

### 3.5 Secondary Outcomes

#### 3.5.1 Pre‐ and Post‐Transplant Dialysis

Pretransplant dialysis within 1 week was more common among high‐MELD recipients (61.3% vs. 14.0%, *p* < 0.001). Post transplant dialysis was more common in the MELD ≥35 group on both day 7 (30.6% vs. 8.0%, *p* < 0.001), and day 28 (20.7% vs. 6.6%, *p* < 0.001).

#### 3.5.2 Intraoperative Blood Product Utilization

There were statistically significant differences in intraoperative transfusion requirements between recipients with MELD ≥35 and those with MELD <35, with a higher volume of all blood products utilized in the MELD ≥35 cohort. (Figure S1 and Table 2).

**TABLE 2:** Peri‐ and postoperative outcomes and laboratory results by MELD ≥35 versus < 35.

	MELD score		
Variable	35 and over	Less than 35	*p*
Biliary Complications, *n* (%)	33 (29.7)	138 (31.6)	0.79
Graft Failure, *n* (%)	31 (27.9)	108 (24.7)	0.57
Death, *n* (%)	17 (15.3)	61 (14.0)	0.83
Readmission, *n* (%)	24 (21.6)	75 (17.2)	0.34
Dialysis, at 7 days postop, *n* (%)	34 (30.6)	35 (8.0)	<0.001
Dialysis, at 28 days postop, *n* (%)	23 (20.7)	29 (6.6)	<0.001
Length of transplant procedure median [IQR], h	6.0 [5.0, 7.0]	6.0 [5.0, 7.0]	0.85
Length of stay in ICU after transplant median [IQR], d	6.0 [4.0, 8.0]	4.0 [3.0, 6.0]	<0.001
Length of hospital stay median [IQR], d	15.0 [10.5, 22.5]	11.5 [8.0, 18.0]	<0.001
PRBC median [IQR], L	2.70 [2.03, 4.36]	2.10 [1.20, 3.30]	<0.001
FFP, median [IQR], L	2.88 [1.62, 5.23]	2.10 [1.16, 3.30]	<0.001
PLTS, median [IQR], L	1.30 [0.70, 1.78]	0.91 [0.57, 1.44]	0.001
Cryo, median [IQR], L	0.31 [0.20, 0.48]	0.22 [0.12, 0.40]	0.017
Clot stiffness, post‐reperfusion (hPa) (median [IQR])	9.0 [6.9, 13.7]	10.8 [8.7, 13.4]	0.11
Heparinase clot time, post‐reperfusion median [IQR], s	161 [152, 169]	160 [148, 172]	0.64
Clot time, post‐reperfusion median [IQR], s	177 [167, 212]	179 [161, 198]	0.27
Clot time ratio, post‐reperfusion median [IQR], s	1.1 [1.1, 1.2]	1.1 [1.0, 1.1]	0.30
PLT‐contribution to clot stiffness, post‐reperfusion, median [IQR], hPa	8.1 [6.5, 12.1]	9.4 [7.7, 12.1]	0.20
FIBR‐contribution to clot stiffness, post‐reperfusion, median [IQR], hPa	1.1 [0.8, 1.5]	1.4 [1.0, 1.7]	0.11
Hemoglobin, 1 day post‐op, median [IQR], g/dL	9.4 [9.0, 10.2]	9.8 [9.1, 10.9]	0.048
Hemoglobin, 30 days post‐op, median [IQR], g/dL	8.1 [7.6, 8.9]	9.0 [8.0, 10.3]	<0.001
Creatinine, 1 day post‐op, median [IQR], g/dL	1.8 [1.4, 2.2]	1.3 [1.0, 1.8]	<0.001
Creatinine, 30 days post‐op, median [IQR], g/dL	1.5 [0.9, 2.4]	1.2 [0.9, 1.6]	0.025
Bilirubin, 1 Day post‐op, median [IQR], g/dL	6.4 [4.1, 8.8]	3.3 [2.0, 5.3]	<0.001
Bilirubin, 30 days post‐op, median [IQR], g/dL	0.9 [0.7, 1.2]	0.7 [0.4, 1.0]	0.002
AST, 1 day post‐op, median [IQR], (U/L)	500 [275, 806]	714 [400, 953]	<0.001
AST, 3 days post‐op, median [IQR], (U/L)	99.5 [68.5, 214]	138 [86.8, 266]	0.013
AST, 7 days post‐op, median [IQR], (U/L)	34.0 [22.0, 48.2]	35.0 [25.0, 53.2]	0.32
AST, 30 days post‐op, median [IQR], (U/L)	18.0 [14.0, 25.5]	18.5 [13.0, 26.0]	0.64
ALT, 1 day post‐op, median [IQR], (U/L)	307 [182, 535]	452 [254, 826]	<0.001
ALT, 3 days post‐op, median [IQR], (U/L)	187 [110, 367]	262 [149, 471]	0.002
ALT, 7 days post‐op, median [IQR], (U/L)	74.0 [46.0, 127]	102 [60.0, 158]	0.001
ALT, 30 days post‐op, median [IQR], (U/L)	20.0 [14.0, 39.0]	20.0 [13.8, 35.0]	0.98
INR, first post‐op reading, median [IQR]	3.1 [2.3, 3.7]	2.5 [2.0, 3.3]	<0.001
INR, 30 days post‐op, median [IQR]	1.4 [1.1, 2.0]	1.3 [1.2, 2.0]	0.99

*Note:* Comparison of peri‐ and postoperative outcomes between high‐ and lower‐MELD liver transplant recipients. Continuous variables are expressed as *median [IQR]* and categorical variables as *n (%)*. Unadjusted *p* values.

Abbreviations: ALT, alanine aminotransferase; AST, aspartate aminotransferase; FFP, fresh frozen plasma; Hgb, hemoglobin; ICU, intensive care unit; INR, international normalized ratio; IQR, interquartile range; MELD, Model for End‐Stage Liver Disease; PLT, platelet; PRBC, packed red blood cells.

#### 3.5.3 Intraoperative Viscoelastic Clotting Parameters

Assessment of viscoelastic coagulation profiles post‐reperfusion demonstrated no significant differences between the MELD ≥35 and MELD <35 groups across all parameters.

#### 3.5.4 Postoperative Laboratory Trends

INR was significantly higher in the MELD ≥35 group at day 1, but the difference was not statistically significant by day 7. AST and ALT were significantly lower in the MELD ≥35 group at day 1, but the difference was no longer significant by day 7 for AST and by day 30 for ALT (Figure S2 and Table 2). Bilirubin and creatinine were significantly higher in the MELD ≥35 group at postoperative day 1 and remained significantly higher by day 30 postoperatively, but did trend toward normal.

#### 3.5.5 Hospital Times and Readmission Rates

Differences in median length of transplant procedure were not statistically significant (6.0 [5.0, 7.0] hours for MELD ≥35 vs. 6.0 [5.0, 7.0] hours for MELD <35, *p* = 0.85), but statistically significant differences were found for median length of ICU stay following transplant (6.0 [4.0, 8.0] days vs. 4.0 [3.0, 6.0] days, *p* < 0.001) and overall length of hospital stay (15.0 [10.5, 22.5] days vs. 11.5 [8.0, 18.0] days, *p* < 0.001). Readmission rates for MELD ≥35 were comparable to MELD <35 (21.6% vs. 17.2%, *p* = 0.34).

#### 3.5.6 Complications

MELD ≥35 35 was not significantly associated with an increased risk of biliary complications in the adjusted Cox regression model (HR: 1.02; 95% CI: 0.65–1.60; *p* = 0.94) (Figure 2). No pVVB catheter‐related complications as defined by insertion site infection, arterial, venous or nerve injury associated with placement or removal of the line, clinically significant bleeding on insertion or removal, pneumothorax, or air embolism were identified.

## 4 Discussion

In this single‐center cohort, we evaluated a modified technique of pVVB in LT recipients stratified by pretransplant severity, defined by MELD ≥35 versus MELD <35. We chose to include all patients in whom pVVB was utilized (high and low MELD), so the technique was familiar to all the LT team members and there was no learning curve when applied to the high MELD patients. Despite the expected increase in perioperative risk among high‐MELD recipients, including greater dialysis exposure, more acute liver failure, and higher preoperative bilirubin, creatinine, and INR levels, no significant differences were observed in key postoperative outcomes. Rates of biliary complications, graft failure, mortality, and readmission were comparable between groups, as were viscoelastic clotting profiles. Importantly, there were no mortalities directly attributable to the use of portal bypass. These findings suggest that with proper experience, pVVB may be safely utilized in high MELD individuals. However, it is important to note that the confidence intervals for biliary complications, graft failure, mortality, and readmission were relatively wide and should therefore be interpreted with caution. This technique was easy to use, is rapid to implement, and was not associated with any complication directly related to the bypass procedure itself.

Internal comparison of pVVB versus non pVVB is not available in this study as all but one transplant surgeons at this program routinely use pVVB in all LT. A comparison between the use of pVVB will therefore introduce significant confounding. Outcomes within the study cohort would predominantly reflect differences between surgeons (technique, case selection, intraoperative decision‐making, experience) rather than the isolated effect of pVVB itself. To investigate whether pVVB itself, independent of surgeon and center practice, improves outcomes in high‐MELD recipients, requires either a multicenter cohort with variation in pVVB use that is not confounded by surgeon, or a prospective randomized or quasi‐randomized design in which eligible high‐MELD patients are allocated to pVVB or no pVVB independent of surgeon preference.

### 4.1 Laboratory Value Trends and Physiologic Recovery

Postoperative laboratory trends mirrored expected recovery kinetics and did not reveal clinically meaningful deterioration in high‐MELD patients. As anticipated, bilirubin and creatinine levels (components of the MELD score) were initially higher among recipients with MELD ≥35, reflecting more severe pretransplant hepatic and renal dysfunction, but both improved by postoperative day 30, indicating satisfactory graft function and renal recovery. Elevated AST and ALT normalized appropriately post transplant with no significant difference between high and low MELD groups. These trends indicate that hepatic recovery in the higher‐MELD cohort was not worse than in the lower‐MELD cohort despite greater baseline severity. While 30 day hemoglobin did differ statistically, the difference of less than 1 point is not clinically significant which supports our claim of safe utilization of pVVB in the high MELD group.

### 4.2 Transfusion Requirements

Intraoperative transfusion requirements were expectedly higher in the high MELD strata. As literature has shown, baseline coagulopathy and greater portal hypertension in the MELD ≥35 group, is likely what is being reflected in the increased intra‐operative blood products used. This has been shown in literature that higher acuity patients experience more bleeding during transplantation. The difference is statistically significant as well as clinically relevant.

### 4.3 Viscoelastic Properties and Coagulation Stability

Viscoelastic testing after reperfusion revealed no statistically significant differences in clotting parameters between groups. Despite baseline hepatic dysfunction, post‐reperfusion clot strength was similar between MELD strata. The data therefore indicates that recipients with MELD ≥35 who received pVVB did not show worse coagulation parameters than lower‐MELD recipients who also received pVVB, consistent with a favorable safety profile for the technique in this population.

### 4.4 Renal Outcomes and Dialysis Trends

Renal outcomes reflected the substantially greater baseline renal dysfunction among recipients with MELD ≥35. Pretransplant dialysis was more common among recipients with MELD ≥35 (61.3% vs. 14%), and these recipients remained more likely to require dialysis at postoperative day 7(30.6% vs. 8.0%) and day 28 (20.7% vs. 6.6%). Within the MELD ≥35, the proportion of recipients receiving dialysis decreased between postoperative days 7 and 28, however, the present analysis did not evaluate patient‐level transitions off dialysis and therefore cannot establish or compare rates of renal recovery between MELD strata. While these patients remained more likely to require dialysis by postoperative days 7 and 28, they also demonstrated a higher rate of renal recovery by day 28.

### 4.5 Teaching Hospital

An important contextual factor is that this study was conducted at a teaching hospital where residents and fellows actively participate in all phases of LT. The use of portal bypass creates a more controlled physiologic and technical environment during the most demanding portions of the operation. By decompressing the splanchnic and systemic venous beds and stabilizing preload during the anhepatic phase, bypass reduces venous congestion and hemodynamic variability, decreasing the chances of encountering large, unexpected blood loss, providing a clearer operative field and more predictable window for complex dissection. Thereby allowing for a more favorable hepatectomy and transplant, highlighting its role as a stabilizing adjunct in training settings.

### 4.6 Potential Clinical Applications of pVVB

This data suggest that the use of pVVB is feasible high MELD individuals. A possible explanation we propose is that, the controlled decompression afforded by VVB may stabilize intraoperative hemodynamics and reduce splanchnic congestion without adversely affecting coagulation or transfusion requirements, however the current study is not able to verify this theory.

From a clinical standpoint, these results challenge the notion that VVB is obsolete in modern LT. While contemporary anesthesia and rapid implantation have minimized the need for routine bypass, this study supports its safety in high MELD recipients; conceivably in cases of severe portal hypertension, physiologic instability, or prolonged anhepatic phase. While the authors of this study implement pVVB routinely in all LT patients, pVVB may be advisable when clamp testing reveals preload intolerance or significant reductions in venous return, when complex or extended hepatectomy/implantation is anticipated (e.g., re‐do transplant, difficult caval dissection, portal vein thrombectomy), or when massive splanchnic engorgement is present‐ exact thresholds for each of the aforementioned scenarios needs to be studied for more specific recommendations. In teaching environments, its use can provide a safer operative context for supervised trainees without compromising graft or patient outcomes. Conversely, in straightforward piggyback cases with stable hemodynamics and short anhepatic time, bypass may be omitted to conserve cost and resources. Ultimately, familiarity with pVVB remains a valuable skill for any transplant surgeon when faced with complex or unstable scenarios.

### 4.7 Possible Failure Modes

Establishing pVVB may not be universally successful. Several failure modes merit discussion for centers considering adoption of this technique. The most common technical obstacle is inadequate portal vein cannulation, encountered in patients with portal vein thrombosis, cavernous transformation, or altered anatomy from prior TIPS placement; in these cases, alternative cannulation sites or abandonment of bypass in favor of proceeding with the standard technique may be required. Inadequate circuit flow despite successful cannulation, due to cannula malposition, kinking, or early thrombus formation, can typically be resolved by cannula repositioning or line exchange, though in a subset of cases this is not correctable intraoperatively and bypass is abandoned. Circuit‐related mechanical failures, including pump malfunction or line disconnection, are managed by emergent circuit exchange when feasible. In our experience, the most common encountered issue, although still quite rare, is cannula kinking which was always able to be addressed by finding the bend or kink in the line and never required portal bypass to be abandoned. These findings underscore that pVVB, while generally reliable, requires a surgical staff prepared to recognize failure early and revert to standard technique without prolonging cross‐clamp or anhepatic time if needed.

### 4.8 Limitations and Future Directions

This retrospective single‐center study carries inherent limitations, including potential selection bias in bypass use and limited control over intraoperative variables. A contemporaneous internal comparison of pVVB versus no pVVB was not feasible because bypass use at our institution is predominantly surgeon‐dependent, with five of six transplant surgeons routinely using pVVB for all LT recipients. Consequently, a pVVB versus no‐pVVB comparison would largely reflect differences between surgeons rather than the isolated effect of the technique. Nevertheless, the consistency of findings across hepatic, biliary, and renal endpoints provides preliminary evidence supporting the safety of pVVB in the high MELD population. A follow up clinical study comparing pVVB and non pVVB would likely be of much interest. Hemodynamic data and pressor requirements were not available. Future multicenter prospective studies employing standardized bypass protocols and detailed hemodynamic monitoring are warranted to validate these findings and refine selection criteria for optimal utilization.

## 5 Conclusion

In this single‐center cohort of LT recipients treated with pVVB, MELD ≥35 was not significantly associated with graft failure, mortality, or biliary complications despite greater baseline acuity. Although pVVB is not commonly used in many major LT centers, we believe that being familiar with its utility and comfortable with its implementation is an important tool to have in a LT surgeon's repertoire. Collectively, these results support the feasibility of pVVB in high‐MELD recipients; whether the technique itself contributes to this parity in outcomes will require comparison with a non‐pVVP cohort.

## Funding

Private grant from Ms. Kristina L. Scheid, Mendota Heights, MN.

## Ethics Statement

The University of Minnesota IRB approved this study. (STUDY00012534).

## Conflicts of Interest

The authors of this manuscript have no conflicts of interest to disclose.

## Supporting information




**Supporting Information**: ctr70703‐sup‐0001‐FiguresS1‐S2.docx

## Data Availability

All data can be made available on request.
