# Supplementary material for: Outcomes of a Modified Portal Venovenous Bypass Technique During Liver Transplantation in High‐MELD Recipients
Source: Clin Transplant. 2026 Sep 29;40(10):e70703. doi: 10.1111/ctr.70703 (PMC13621918; doi:10.1111/ctr.70703)
Supplement: Supplementary file 1 [file CTR-40-e70703-s001.docx]

**Supplemental Figure 1**

**Supplemental Figure 1.** Intraoperative blood product utilization and viscoelastic profiles

A: Intraoperative transfusion requirements of packed red blood cells (PRBC), fresh frozen plasma (FFP), platelets (PLT), and cryoprecipitate (Cryo). B: Post-reperfusion viscoelastic parameters including clot stiffness, clot time, platelet- and fibrin-derived contributions.

**Abbreviations:** MELD, Model for End-Stage Liver Disease; FIBR, fibrinogen; PLT, platelet

**Supplemental Figure 2**

**Supplemental Figure 2.** Temporal trends in laboratory parameters after liver transplantation

Postoperative trends in (A): AST, (B): serum bilirubin, (C): creatinine, and (D): INR over postoperative days 1, 3, 7, and 30, stratified by MELD ≥35 and MELD <35. Data presented as median values with 25th and 75th percentiles representing the upper and lower boundaries.

**Abbreviations:** AST, aspartate aminotransferase; Hgb, hemoglobin; INR, international normalized ratio; MELD, Model for End-Stage Liver Disease.
